# What saccadic eye movements tell us about TMS-induced neuromodulation of the DLPFC and mood changes: a pilot study in bipolar disorders

**DOI:** 10.3389/fnint.2014.00065

**Published:** 2014-08-19

**Authors:** Lysianne Beynel, Alan Chauvin, Nathalie Guyader, Sylvain Harquel, David Szekely, Thierry Bougerol, Christian Marendaz

**Affiliations:** ^1^Department of Psychology, Laboratory of Psychology and Neurocognition, Grenoble Alpes University, Université Pierre Mendes FranceGrenoble, France; ^2^Department of Images and Signal, Grenoble Image Parole et Signal Automatique-Lab, Grenoble Alpes University, St Martin d’HéresGrenoble, France; ^3^Department of Psychology, IRMaGe, Grenoble Alpes UniversityGrenoble, France; ^4^Department of Psychiatry and Neurology, Hospital of Grenoble, Grenoble Alpes UniversityLa Tronche, France

**Keywords:** antisaccades, DLPFC, rTMS iTBS, bipolar disorder, short-term neuromodulation, long-term neuromodulation

## Abstract

The study assumed that the antisaccade (AS) task is a relevant psychophysical tool to assess (i) short-term neuromodulation of the dorsolateral prefrontal cortex (DLPFC) induced by intermittent theta burst stimulation (iTBS); and (ii) mood change occurring during the course of the treatment. Saccadic inhibition is known to strongly involve the DLPFC, whose neuromodulation with iTBS requires less stimulation time and lower stimulation intensity, as well as results in longer aftereffects than the conventional repetitive transcranial magnetic stimulation (rTMS). Active or sham iTBS was applied every day for 3 weeks over the left DLPFC of 12 drug-resistant bipolar depressed patients. To assess the iTBS-induced short-term neuromodulation, the saccadic task was performed just before (S1) and just after (S2) the iTBS session, the first day of each week. Mood was evaluated through Montgomery and Asberg Depression Rating Scale (MADRS) scores and the difference in scores between the beginning and the end of treatment was correlated with AS performance change between these two periods. As expected, only patients from the active group improved their performance from S1 to S2 and mood improvement was significantly correlated with AS performance improvement. In addition, the AS task also discriminated depressive bipolar patients from healthy control subjects. Therefore, the AS task could be a relevant and useful tool for clinicians to assess if the Transcranial magnetic stimulation (TMS)-induced short-term neuromodulation of the DLPFC occurs as well as a “trait vs. state” objective marker of depressive mood disorder.

## Introduction

### rTMS treatment of depression: necessity to assess the induced neuromodulation

Transcranial magnetic stimulation (TMS) is a non-invasive technique that induces a magnetic field on the skull which changes rapidly enough to induce electrical currents in underlying cortical tissue and thus to induce a neuromodulation effect (Hallet, 2000). Repeated TMS (rTMS) has been used as a therapeutic tool for the treatment of drug-resistant mood disorders since the 1990s; patients receive a daily dose of rTMS over frontal regions for several weeks. The rationale for this treatment is that whereas a single rTMS session induces an early long-term potentiation of the targeted cortical area (short-term neuromodulation), cumulative rTMS sessions induce widespread late long-term potentiation across multiple neural circuits (Noda et al., 2013).

Meta-analyses of rTMS as a treatment for depression have been recently published (Janicak et al., 2010; Slotema et al., 2010; George and Post, 2011; Berlim et al., 2014). All these studies agree that the therapeutic efficacy of rTMS is statistically significant but affects few patients. For example, Berlim et al. (2014) showed that about 30% of depressed patients receiving active excitatory rTMS responded to the treatment compared to 10% of patients who received sham treatment (analysis based on 29 studies using randomized, double-blind and sham-controlled trials).

To improve the therapeutic efficacy of rTMS, a new protocol—theta burst stimulation (TBS)—(Huang et al., 2005) has been developed. TBS has been shown not to differ from rTMS in terms of strength and direction of aftereffects (Thut and Pascual-Leone, 2010; Di Lazzaro et al., 2011) but to exert longer-lasting post-stimulation effects (Thut and Pascual-Leone, 2010) with less stimulation time and lower stimulation intensity (Hinder et al., 2014). For these reasons TBS is of particular interest for clinicians, and recently some studies investigated the therapeutic effectiveness of TBS for the treatment of mood disorders, using intermittent theta burst stimulation (iTBS) that produces long-term potentiation-like effects, or continuous TBS (cTBS) that produces long-term depression-like effects. Li et al. (2014) compared cTBS, iTBS and sham stimulation. They found that depressive mood improved in all groups, with a better antidepressant effect for iTBS (40% responders) than for cTBS (25% responders) and sham (13% responders). Plewnia et al. (2014) compared TBS to sham stimulation. They found that 56% of patients receiving active TBS responded to the treatment compared to 25% of patients who received sham treatment. As a whole, these results indicate that whatever the TMS protocol, the therapeutic efficacy of rTMS is statistically significant but remains limited, and the rTMS clinical relevance is still debated (Padberg and George, 2009).

Apart from idiopathic reasons, several technical/neuro­physiologic factors might account for the lack of TMS efficacy. For example, the stimulation parameters (intensity, frequency) might not be adjusted properly for every patient, the dorsolateral prefrontal cortex (DLPFC) might be identified or targeted incorrectly (Fox et al., 2012), or the TMS treatment might fail to induce short-term neuromodulation of the DLPFC in some patients, which prevents the long-term potentiation. To move forward on these issues, it is crucial to ensure that TMS-induced neuromodulation of the DLPFC effectively occurs. This question requires the development of an instrument that can objectively assess this neuromodulation. To be helpful for clinicians, this instrument has to be non-invasive and easy to use. We assumed that an oculometric task such as the antisaccade (AS) task may provide such a tool (Crevits et al., 2005; Malsert et al., 2012a,b, 2013).

### Relevance of AS task to assess TMS-induced neuromodulation of the DLPFC and of mood improvement

To perform an AS requires the inhibition of a reflexive saccade toward a target and the generation of a voluntary saccade in the opposite direction (Everling and Fischer, 1998). Several lines of evidence suggest that the DLPFC is involved in a cortical network underlying the inhibition process required to perform a correct AS. Some studies showed that lesions of the DLPFC in humans lead to an increase in inhibition errors during AS tasks (Pierrot-Deseilligny et al., 2003; Ploner et al., 2005). Electrophysiological and TMS studies confirmed the involvement of the DLPFC in saccadic inhibition, suggesting a lateralized inhibitory control of the DLPFC (Müri et al., 1999; Johnston and Everling, 2006; Wegener et al., 2008; Müri and Nyffeler, 2008; but Nyffeler et al., 2007). As a consequence, studying AS performance during rTMS treatment applied over the DLPFC, might inform about the rTMS-induced neuromodulation of this cortical region. Moreover, some studies showed that a clinical improvement could result in cognitive improvement. Biringer et al. (2005) showed that recovery from depression is associated with a recovery of many aspects of executive functions to a normal level. Moreover, using rTMS over the left DLPFC on treatment-resistant depressed patients, Kedzior et al. (2012) found, after 20 days of rTMS treatment, a cognitive improvement for these patients as well as a mood improvement. As a consequence, we expected that the saccadic task could also be a marker of mood improvement.

### Pilot study

To test the relevance of AS task to assess TMS-induced neuromodulation of the DLPFC and of mood improvement, we conducted a pilot study in 12 drug-resistant bipolar depressed patients receiving either active or sham iTBS over the left DLPFC for 1–3 weeks. Short-term TMS-induced neuromodulation was tested by comparing performances to a saccadic task performed just before and just after the iTBS session. It was expected that AS performances would be better just after the iTBS session than before for the iTBS active group and not for the sham group; and that changes in AS performances would be stronger in the contralateral hemifield of the stimulated DLPFC, i.e., here, the right hemifield. The relevance of the AS task to assess patients’ mood improvement was analyzed by computing the correlation between mood improvement and AS performance improvement. Besides this, we examined the difference between depressed bipolar patients and healthy controls; it was expected that before treatment, patients’ AS performances would be impaired compared to those of healthy subjects.

## Methods

### Experimental design

**Figure 1:**
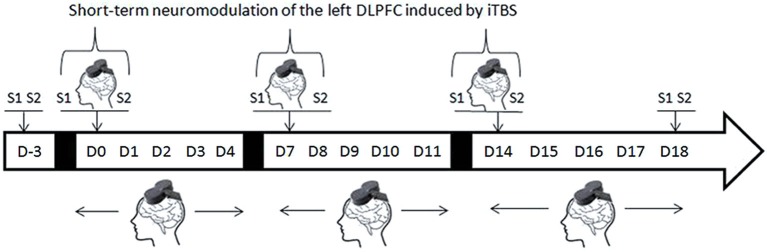
**Experimental design (D = day, S1 and S2 = first and second AS task sessions)**. On D-3 two AS task sessions are performed for training purposes. Repetitive TMS treatment was administered every day during the week but AS task sessions were performed only on the first day of each week, immediately before (S1) or immediately after (S2) rTMS treatment.

Active or sham iTBS treatment was applied twice a day, 5 days a week for 1–3 weeks. Two sessions of saccadic task were conducted on the first day of each week (D0, D7, and D14), one session (S1) before the iTBS and a second (S2) immediately following the iTBS (Figure 1). To ensure that any improvement in the AS performances could not be accounted for by a simple learning effect, two training sessions were administered 4 days before (D-3) the beginning of the iTBS treatment to minimize the practice-related effects. Two sessions of the saccadic task were also performed at the end of the protocol (D18).

### Subjects

#### Patient group

Twelve patients (6 females, 6 males; mean age 51.6 ± 11.7 years) with drug-resistant bipolar disorder (Types I, II, or III) participated in this randomized double-blind placebo-controlled iTBS study. The study was approved by the regional Ethics Committee (Committee for the Protection of Persons in biomedical researches: CPP Sud-Est VI) and allowed by the ANSM (French National Agency for Medicines and Health Products Safety) (Authorization: 2010-A01085-34).

Patients were randomly assigned to the active or to the sham group. This randomization was performed using a randomization table and assessed 3 by 3 to have the same proportion of patients in each group. As allowed by the protocol of the study, the TMS operator did the un-blinding for the purpose of this study, independently from the clinical research team, who remained fully blind of each patient’s treatment status.

The inclusion criteria were having a drug-resistant major depressive episode defined according to DSM IV-TR. The criterion of severity was a score of over 20 with a maximum of 60 on the *Montgomery and Asberg Depression Rating Scale* (MADRS: Montgomery and Asberg, 1979) (see Table 1 for details). Drug resistance was defined as the absence of a response to any antidepressant treatment over at least a 4-week period of time. Patients with a history of substance abuse and patients who did not meet inclusion criteria for use of TMS and MRI (no pacemaker, no history of epilepsy or other neurological disorders) were excluded. Mood-stabilizers were allowed during the treatment period only if the patients were stable for at least 4 weeks before the rTMS treatment. Only anxiolytic drugs (cyamemazine or hydroxyzine) at low doses could be administered if necessary during the rTMS treatment period.

**Table 1:** **Individual MADRS scores arranged by Group (active vs. sham) and day of treatment (beginning vs. end) and intergroup comparisons**.

Subjects	Group	MADRS beginning	MADRS end	Improvement
1	Active	30	8	73.3%
2	Active	36	13	63.9%
3	Active	38	13	65.8%
4	Active	32	23	28.1%
5	Active	24	8	66.7%
6	Sham	24	5	79.2%
7	Sham	25	16	36%
8	Sham	37	9	75.7%
9	Sham	27	4	85.2%
10	Sham	32	11	65.6%
11	Sham	38	36	5%
12	Sham	25	14	44%
**Inter-Group Comparisons**		
	**(m ± SD)**	***t* and *p*-values**
Active vs. Sham (beginning)	(32 ± 5 vs. 30 ± 6)	0.68 (0.51)
Active vs. Sham (end)	(13 ± 6 vs. 14 ± 11)	−0.10 (0.92)
Active vs. Sham (improvement)	(60 ± 18 vs. 56 ± 29)	0.25 (0.81)

#### Control group

Twelve control subjects (7 females, 5 males; mean age 50.6 ± 10.9 years) also participated in the study. These subjects had no psychiatric history and were not taking any medication. This group only performed one saccadic task session. Every participant provided written, informed consent.

### Intermittent theta burst stimulation (iTBS)

The iTBS was applied using a figure-of-eight coil (MCF-B65-H0) and an air-cooled stimulator (MagProX100, MagVenture). For each patient the left DLPFC was delimited using three-dimensional magnetic resonance imaging (3D-MRI). The TMS coil was monitored throughout stimulations using a neuronavigation device (TMS Navigator, Localite). We used an iTBS protocol in which a 2 s train of bursts containing three pulses at 50 Hz was repeated at 200 ms (i.e., 5 Hz) every 10 s (Huang et al., 2005), these parameters mimicked the theta rhythm in EEG nomenclature. The TMS operators applied the iTBS twice per day, with a minimum inter-session interval of 3 h every day for 1–3 weeks depending of clinical relevance. For each rTMS session 990 pulses were administered to give a total of 5 min 30 s of stimulation, thus, patients received 1980 pulses per day. Patients were stimulated with either an active coil (active group: *n* = 5) or a sham coil (sham group: *n* = 7). The sham coil made the same “clicking” sound than the active coil, and produced a weak magnetic field on the scalp for reproducing the same skin sensation than the active coil.

We individually set the stimulation intensity at 80% of the patient’s resting motor threshold (RMT), which we determined 3 days before the beginning of the iTBS treatment phase (D-3). We began by placing three electrodes over the patient’s first dorsal interosseous muscle (FDI) in a belly-tendon montage. Electromyograms were amplified (1–10 K), band-pass filtered (1–6 KHz), and sampled at 12 KHz using a Dantec Keypoint portable system (Natus Medical Incorporated). We placed the coil on the “hotspot”: the position on the motor cortex that elicited the greatest motor-evoked potential (MEP) in the contralateral FDI. We defined the RMT as the minimum stimulation intensity needed to evoke a MEP greater than 50 μV on at least 5 out of 10 consecutive trials (Rossini et al., 1994).

### Saccadic task

We used an EyeLink 1000 video-based eye-tracking system (SR Research) with a temporal resolution of 500 Hz. The eye-tracker detects saccades automatically, using three thresholds: velocity (30°/s), acceleration (8000°/s2), and saccadic motion (0.15°). Stimuli were displayed on a computer screen located 57 cm from the participants. The computer screen resolution was 1024 × 768 pixels and the screen refresh rate was 85 Hz. Participants were seated in a darkened room and their heads were stabilized using chin rests. We used a SPAN task (Saccade: Pro, Anti and No) which mixed three types of saccades: antisaccades (AS), no-saccades (NS) and prosaccades (PS). PS was used to verify that the bipolar patients did not suffer from a general deficit in saccadic function. Additionally, compared to a traditional AS task, this mixed saccadic paradigm increased the cognitive load in terms of executive functions, and thus reinforced the implication of the DLPFC (Smith and Jonides, 1999).

**Figure 2:**
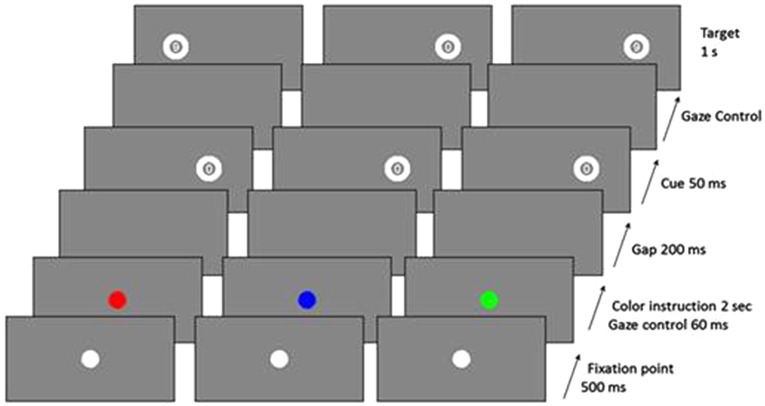
**Examples of trial sequences for the three trial types (red = AS, blue = NS, green = PS) (from Malsert et al., 2013)**.

Each trial began with a 500 ms presentation of a white central fixation dot, and then the central fixation dot became red, blue, or green for 2 s. Participants were told to make a PS if it was green, an AS if it was red, or an NS response if it was blue (Figure 2). After this time, a blank screen was displayed for a 200 ms gap, and a “cue”, the number “0”, was flashed for 50 ms at 10° peripherally (randomly on the right or left side of the screen). During AS trials, patients had to look towards the opposite side from the cue as quickly as possible in order to identify a numeric target (“6” or “9”), which was presented for 1 s beginning as soon as they looked at the correct location (gaze-contingent display) or after a 2-s delay. During PS trials, patients had to look as quickly as possible towards the side of the cue to identify the numeric target. During NS trials, they had to keep their gaze fixed on the center of the screen. There was a break of 1 s between two successive trials. During the first SPAN session, both patients and healthy controls received 20 practice trials and 80 test trials (16 NS trials, 32 PS trials, and 32 AS trials). In the following SPAN sessions, patients performed 80 test trials. We assessed the performances using the inhibition error rate, i.e., the proportion of saccades towards the cue for AS and NS trials and the latency of correct saccades for PS and AS trials.

We analyzed the oculometric performance, i.e., the inhibition error rates and the saccadic reaction times using Matlab (MATLAB, R2009b, The MathWorks Inc., Natick, MA, 2009) and Statistica (Statistica 10, Statsoft Inc., 1984).

### Mood evaluation

We analyzed MADRS scores at the beginning and at the end of the experiment in order to assess mood changes over the time course of the experiment, as well as to assess responses to the iTBS treatment. We defined “response to the treatment” when there was a 50 % improvement of the MADRS score and “remission” when the MADRS score became lower than 8.

## Results

In the following results we only present the inhibition error rates in AS trials as we did not find any differences for PS, NS and latencies of AS trials.

### Comparison of “healthy subjects vs. depressive bipolar patients”

We performed an ANOVA on inhibition error rates with the Group (bipolar patients (*n* = 12) and healthy controls (*n* = 12)) as the between-subjects factor, and the Cue Position (left or right) as the within-subjects factor. This comparison was performed on the learning session only (D-3) for both groups. The ANOVA revealed a main effect of the Group (*F*_(1,22)_ = 4.8; *p* = 0.04). Patients committed significantly more errors than did controls (26.9% vs. 13.8%). We did not find any effect of the Cue Position, nor of the interaction Group × Cue Position (*F*_(1,22)_ < 1).

### Short-term iTBS neuromodulation

**Figure 3:**
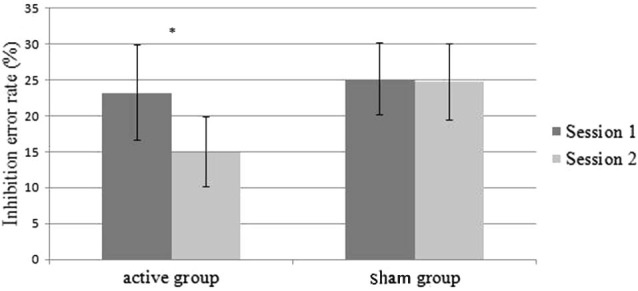
**Mean inhibition error rates on the AS task as a function of the sessions (session 1 vs. session 2) and group (active vs. sham)**. Errors bars represent standard error.

We performed another ANOVA on inhibition error rates with the Group” (active (*n* = 5) and sham (*n* = 7)) as the between-subjects factor, and the Session (Session 1 and Session 2) as the within-subjects factor. The ANOVA did not reveal any effect of the Group (*F*_(1,10)_ < 1). However, we found a main effect of the Session (*F*_(1,10)_ = 6.29; *p* = 0.03). Performances were improved in session 2 compared to session 1 (24.2% vs. 19.9%). We also found a significant interaction Group × Session (*F*_(1,10)_ = 5.29; *p* = 0.04) (Figure 3). Patients in the active group committed fewer errors in session 2 than in session 1 (23.2% vs. 14.9%) (*F*_(1,10)_ = 9.9; *p* = 0.01), while the performances of the patients in the sham group did not show any improvements (25.1% vs. 24.8%) (*F*_(1,10)_ < 1) (Figure 3). When the cue was presented in the right hemifield (contralateral field of iTBS neuromodulation), 100% of patients in the active group improved their performances (vs. 57% of the sham group).

### Effects of mood improvement

**Figure 4:**
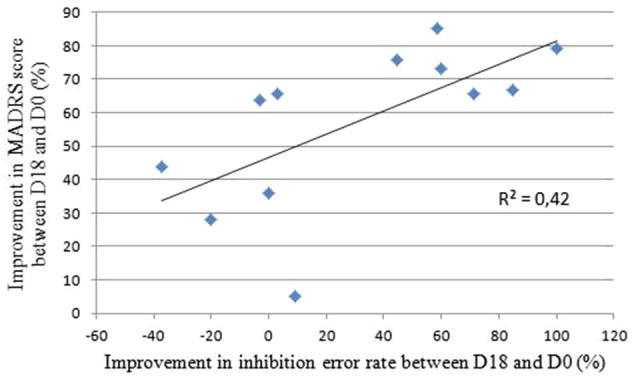
**Correlation between the improvements in MADRS scores and inhibition error rate between the end and the beginning of the rTMS treatment.** Bravais-Pearson coefficient correlation coefficient equals *R* = 0.65, *p* = 0.02 and *R*^2^ = 0.42, where *R*^2^ is the proportion of variance explained by the regression model.

The MADRS scores analyses did not reveal any differences between the active and sham groups before the treatment (*p* = 0.51). After the treatment, four out of five patients in the active group and four out of seven in the sham group responded to the treatment i.e., showed an improvement of more than 50% on the MADRS scores. The *t*-tests did not reveal any differences between groups, neither on the MADRS scores nor for mood improvement (Table 1). To assess the relevance of the AS task as a marker of this mood improvement, we calculated the Pearson’s correlation coefficient between the improvement in the MADRS scores and the difference in inhibition error rates between the end and the beginning of the treatment. We found a significant and positive linear association (*r* = 0.65; *p* = 0.02; *R^2^* = 0.42): better mood is associated with better performance in AS (Figure 4).

## Discussion

This study investigated the AS task as a psychophysical tool to assess the short-term neuromodulation that has been hypothesized to be induced by daily iTBS delivered over the left DLPFC. We also examined the ability of the AS task to discriminate depressive bipolar patients from healthy subjects, and to be a marker of mood improvement.

### Relevance of AS task to discriminate depressed bipolar patients from healthy subjects

Over the past three decades, there has been an increase in the number of neuropsychophysical studies of saccadic performance in psychiatric patient groups (Gooding and Basso, 2008). Some authors suggested that performance on the AS task could be used as a psychophysical marker for mood disorders (García-Blanco et al., 2013; Malsert et al., 2013). Our results confirmed that the AS task could discriminate depressed bipolar patients from healthy subjects. We did not find any group differences on PS performance, which means that depressive bipolar patients did not suffer from a general impairment in saccadic function.

Maybe due to the small number of patients, our study did not show group differences associated with the cue position, i.e., no cerebral asymmetry. Whether depressed bipolar patients display a cerebral asymmetry in the inhibitory functions of the DLPFC is still being discussed in the literature (Clark et al., 2006; Savitz and Drevets, 2009); it is a crucial issue since rTMS therapy is often based on the premise that left DLPFC is hypoactive in depression.

### Relevance of AS task to assess short-term iTBS-induced neuromodulation of the DLPFC

Until now only one study investigated the AS performance during rTMS treatment applied over the DLPFC to investigate the rTMS neuromodulation effect of this cortical region (Crevits et al., 2005). In their study, the left DLPFC of 11 depressed patients was stimulated with 1000 pulses per day of facilitatory rTMS (10 Hz). The treatment lasted for at least 10 sessions, with no more than one session a day, over a maximum period of 3 weeks. The AS task was only performed twice: before the first rTMS session and after the last rTMS session. They found a significant decrease in AS latencies at the end of the treatment. However, the absence of a control group of patients receiving sham rTMS prevented the authors from drawing any conclusions about the long-term effects of rTMS. Additionally, the latency reduction in AS might have reflected a practice effect as the AS task was being repeated at the end of the rTMS treatment. Chauvin et al. (2011) studied the evolution of performance across several sessions of an AS task and found that performance only improved (with decreases in AS latency and inhibition error rate) over the two first sessions. Finally, mood improvement was able to explain the AS performance improvement (Salvadore et al., 2011).

In our knowledge, our study was the first research investigating by oculometry the short-term iTBS-induced neuromodulation of the DLPFC, using a neuronavigation system. As expected, we observed an aftereffect of the iTBS sessions: only the patients in the active group improved their capacity to inhibit the reflexive saccades immediately after the iTBS sessions. This improvement was consistently observed when the cue was presented in the right hemifield, i.e., processed by the left DLPFC. These results provide evidence that iTBS induces short-term neuromodulation of the targeted cortical area. This means that the AS task could be a useful instrument to ascertain whether short-term neuromodulation induced by iTBS occurs.

### Relevance of AS Task as a potential marker of mood improvement

We found that the active and sham groups showed a similar mood improvement. This improvement in the sham group and possibly in the active group too was due to a placebo effect (see Mayberg et al., 2002). Some studies showed that mood improvement could result in cognitive improvement. Biringer et al. (2005) showed that recovery from major unipolar depression is associated with a recovery of many aspects of executive functioning, improving executive functioning to a normal level. In accordance with these studies, we found a significant correlation between mood improvement and AS improvement. This indicates that the saccadic task might not only be a useful marker of the short-term neuromodulation, but a marker of mood changes too.

## Conclusion

This pilot study investigated the iTBS-induced short-term neuromodulation of the DLPFC. This is a crucial issue since little is known about the aftereffect of TBS over the DLPFC while being used in clinical research, in particular, with psychiatric disorders (Soekadar et al., 2009; Chistyakov et al., 2010; Holzer and Padberg, 2010; Plewnia et al., 2014). Exploring the rTMS/iTBS aftereffects requires the development of an instrument to enable one to objectively measure the short-term TBS-induced neuromodulation, which is the *sine qua non* condition to long-term neuromodulation taking place (Pascual-Leone et al., 1994; Valero-Cabré et al., 2011). Our study demonstrates that an AS task could be used to assess it. Moreover, we confirmed that AS performance could discriminate depressive bipolar patients from healthy subjects and be used as a marker of mood variation (response to treatment or relapse into illness) (Malsert et al., 2012b; Aleman, 2013).

However, due to the small sample size, our findings should be replicated using a larger cohort of patients. Moreover, the oculometric task could be improved by adding an emotional component to increase the load imposed on the DLPFC inhibitory control. Indeed, in humans, the existence of connections between the DLPFC and the limbic regions is well established, although the anatomical details of the connections remain unclear in humans (Fox et al., 2012) and monkeys (Petrides and Pandya, 1999). Adding emotional cues should improve the psychometric relevance of the oculometric sessions (García-Blanco et al., 2013). Using an implicit emotional oculometric paradigm every day with depressive bipolar patients should enable a finer-grained analysis of short-term neuromodulation induced by rTMS over the DLPFC and could be a way to optimize and customize the TMS treatment by adjusting rTMS parameters of each patient according to the obtained post-effect.

## Author and contributors

Alan Chauvin, Nathalie Guyader, and Christian Marendaz designed the research; Thierry Bougerol and David Szekely analyzed and interpreted clinical data; Lysianne Beynel, Alan Chauvin, Nathalie Guyader, Sylvain Harquel and Christian Marendaz performed research; Lysianne Beynel, Alan Chauvin, Nathalie Guyader, Sylvain Harquel and Christian Marendaz analyzed and interpreted data; Lysianne Beynel, Alan Chauvin, Nathalie Guyader, Sylvain Harquel, Christian Marendaz and Thierry Bougerol wrote the paper; Lysianne Beynel, Alan Chauvin, Nathalie Guyader, Sylvain Harquel, Christian Marendaz and Thierry Bougerol gave the final approval of the version to be published.

Lysianne Beynel, Alan Chauvin, Nathalie Guyader, Sylvain Harquel, Christian Marendaz, David Szekely and Thierry Bougerol agree to be accountable for all aspects of the work in ensuring that questions related to the accuracy or integrity of any part of the work are appropriately investigated and resolved.

## Conflict of interest statement

The authors declare that the research was conducted in the absence of any commercial or financial relationships that could be construed as a potential conflict of interest.
